# Are conspecific social videos rewarding to chimpanzees (*Pan troglodytes*)? A test of the social motivation theory

**DOI:** 10.1371/journal.pone.0259941

**Published:** 2021-11-24

**Authors:** Michele M. Mulholland, Sarah J. Neal Webb, Mary Catherine Mareno, Kenneth G. Schweller, Steven J. Schapiro, William D. Hopkins

**Affiliations:** 1 Department of Comparative Medicine, The University of Texas MD Anderson Cancer Center, Bastrop, TX, United States of America; 2 Buena Vista University, Storm Lake, IA, United States of America; 3 Department of Experimental Medicine, University of Copenhagen, Storm Lake, IA, United States of America; Universidade de São paulo, BRAZIL

## Abstract

Many claim that social stimuli are rewarding to primates, but few, if any, studies have explicitly demonstrated their reward value. Here, we examined whether chimpanzees would produce overt responses for the opportunity to view conspecific social, compared to dynamic (video: Experiment 1) and static (picture: Experiment 2) control content. We also explored the relationships between variation in social reward and social behavior and cognition. We provided captive chimpanzees with access to a touchscreen during four, one-hour sessions (two ‘conspecific social’ and two ‘control’). The sessions consisted of ten, 15-second videos (or pictures in Experiment 2) of either chimpanzees engaging in a variety of behaviors (social condition) or vehicles, humans, or other animals engaged in some activity (control condition). For each chimpanzee, we recorded the number of responses to the touchscreen and the frequency of watching the stimuli. Independent *t*-tests revealed no sex or rearing differences in touching and watching the social or control videos (*p>*0.05). Repeated measures ANOVAs showed chimpanzees touched and watched the screen significantly more often during the social compared to control video sessions. Furthermore, although chimpanzees did not touch the screen more often during social than control picture sessions in Experiment 2, they did watch the screen more often. Additionally, chimpanzees that previously performed better on a task of social cognition and engaged in more affiliative behavior watched a higher percentage of social videos during the touchscreen task. These results are consistent with the social motivation theory, and indicate social stimuli are intrinsically rewarding, as chimpanzees made more overt responses for the opportunity to view conspecific social, compared to control, content.

## Introduction

According to the social motivation theory, social species are predisposed to orient toward social situations and circumstances, which ultimately increases individual fitness within social environments [see details in 1]. This social motivation is driven by proximate biological mechanisms that make social circumstances (e.g., social relationships, collaborative environments, faces, eye contact) innately rewarding through neurobiological mechanisms in the brain [1, 2]. As such, many social species spend considerable amounts of energy seeking out and orienting toward social stimuli, precisely because these stimuli are innately rewarding [1–3].

The social motivation theory is comprised of three components or processes: 1) social orienting, or attention toward social stimuli; 2) social reward, or seeking out social stimuli due to their innate reward value; and 3) social maintenance, whereby individuals expend energy to engage with others in order to establish, maintain, and enhance relationships [1]. Like humans, nonhuman primates are responsive to social stimuli. Nonhuman primates orient toward and respond to social stimuli across a variety of formats, including static, moving, and live stimuli, demonstrating that such stimuli are highly salient [4–6]. Chimpanzees are no exception, as attention toward social stimuli begins in infancy. For example, chimpanzee neonatal orientation to social stimuli (species-typical sounds and faces) is indistinguishable from that of human neonates [7]. Chimpanzees also extract social signals and information from conspecific auditory cues (i.e., agonistic vocalizations of other chimpanzees). Human and nonhuman primates also engage in a variety of social interactions that are likely mediated by reward contingencies that follow specific types of interactions. For example, mutual grooming may induce positive hedonic responses for both initiator and receiver of the grooming event, which would increase the probability of these individuals grooming again [8, 9]. In short, evidence exists demonstrating that at least the social orientation and social maintenance tenets of the social motivation hypothesis are supported across multiple modalities and species of nonhuman primates.

Less clear from the existing literature in nonhuman primates is the salience and reward value of social stimuli [4], specifically in the context of their use in more abstract two-dimensional modalities of presentation, notably on computer monitors or televisions [10]. For example, social stimuli, such as pictures of faces, have been used in a myriad of studies examining the perceptual, cognitive, and neural systems underlying discrimination of individual and species-specific face processing in nonhuman primates [e.g., 4, 5, 10–14]. Recently, Rossion and Taubert [14] have suggested that, though it is clear that nonhuman primates discriminate between face and non-face stimuli presented on a computer monitor, these findings, in and of themselves, do not demonstrate that nonhuman primates see the images on the screen as social stimuli per se. On the other hand, Putnam, Roman, Zimmerman, and Gothard [13] showed that male rhesus monkeys attempt to interact with conspecifics in videos by gaze following, and that intranasal oxytocin increases this behavior. Additionally, adult male rhesus macaques showed increased visual attention and sympathetic arousal in response to videos with social (conspecifics) compared to nonsocial (nature) content [15]. However, although the use of television or related audio-visual stimulation is often used as a form of enrichment for nonhuman primates (and even more recently for observational learning) [16–20], there is very limited evidence that nonhuman primates would choose to play videos if given a choice [11, 12]. In addition, there is little evidence to suggest that watching television or videos, regardless of the content, has any reward value to nonhuman primates (i.e., can be used as a positive reinforcer to increase or strengthen a particular behavior). There have been several attempts to use access to pictures or videos of social content as a reward in standard learning paradigms and these have produced mixed results. For example, Andrews and Rosenblum [5] trained bonnet macaques on a computerized psychomotor task using food pellets or a live video stream of other monkeys as the reward, and found no significant difference in the number of trials performed over a two-week test period (suggesting the live video stream was as rewarding as the food). In a later study, however, bonnet macaques chose videos of conspecifics over food as a reward [21], indicating that social videos may have a higher reward value than food. In contrast, Washburn and Hopkins [22] found that macaque performance and the number of trials on several computerized automated cognitive tasks were significantly lower when videos were used as the reward compared to food pellets. Furthermore, in a study by Harris et al. [23], only two macaques were able to learn to press a lever when the reward was videos of conspecifics. More recently, Gray et al. [24] compared the reward value of juice and videos in macaques when performing an automated cognitive testing task, and found little evidence that videos of other monkeys were rewarding to their subjects.

A number of studies have demonstrated that chimpanzees will both initiate and respond to joint attention cues, such as gaze direction and pointing [25–28]. Some have suggested that initiation of and response to joint attention cues can be divided into two domains: imperative and declarative [29, 30]. Imperative joint attention is motivated by the subject wanting or requesting something (i.e., food or some other type of object). In contrast, declarative joint attention is motivated by the desire to share attention (without requesting/wanting), and some have suggested that social reward governs the acquisition and use of declarative joint attention in developing children [31, 32], and accounts for phylogenetic differences in joint attention between humans and nonhuman primates [30, 33]. Declarative pointing has rarely been reported in nonhuman primates (including chimpanzees); however, data show they respond to declarative communication cues, including data previously published for a number of the chimpanzees in this study [34]. According to the social motivation theory, differences in performance on such socio-communicative tasks (e.g., joint attention) are likely related to variations in social behavior and cognition [1].

In the current study, we tested the reward value of conspecific social videos and pictures presented during a simple computerized task in a sample of chimpanzees. We defined reward in terms of operant conditioning [35]: the ability of a video/picture to reinforce or increase a particular behavior (in this case, the frequency of touching the screen). We gave chimpanzees access to a touchscreen with a neutral stimulus on the screen that they could touch to initiate the presentation of 15-second video clips of either conspecific social or control content. Of specific interest was the frequency of touching the neutral stimulus to view either the social or control content. Given the basic premise of operant conditioning (that behaviors followed by rewarding events will increase in their probability of occurrence [35]), evidence of increased responding (i.e., the frequency of touching the screen) when rewarded with conspecific social, compared to control, videos would suggest that the social videos are indeed more rewarding. In other words, videos with conspecific social content are rewards that elicit increases in touching behavior compared to videos without such conspecific social content. We tested the chimpanzees twice on each type of video (social and control) to test for a decrease in response due to repeated presentation (i.e., habituation). Responses to videos with greater reward value should not diminish significantly with repeated presentation; therefore, we further hypothesized that chimpanzees would show less habituation to social videos compared to control videos. In other words, there would be little to no decrease in responses over consecutive presentations of social compared to control rewards. Additionally, rearing and sex can impact social behavior and responses to social content. For example, nursery-reared primates may show some deficits in social responsiveness [36], and female primates may have stronger affective responses to social stimuli [37]. Therefore, we examined the effects of rearing and sex on responses in the social and control conditions.

In addition to testing for the reward value of conspecific social videos, we examined whether chimpanzees that were higher or lower in social motivation (i.e., watched and exerted more effort to play conspecific social videos) also differed in their social behavior and cognition. We took advantage of the existence of archival data to test for associations with the reward value of social videos assessed from the touchscreen video task. Based on the social motivation theory, we predicted that variation in watching and initiating playback of the conspecific social videos (i.e., social motivation) would be associated with previously collected measures of social cognition and behavior. Specifically, chimpanzees with poorer performance on a receptive joint attention task, and/or lower levels of overall affiliative social behavior, would have lower social motivation as measured by the touchscreen task.

## Experiment 1 methods

### Subjects

Subjects included 85 chimpanzees from the National Center for Chimpanzee Care at The University of Texas MD Anderson Cancer Center in Bastrop, Texas. All chimpanzees were housed in indoor/outdoor corrals or Primadomes^TM^, with 24-hour access to both areas, except during cleaning. The enclosures contained climbing structures, bedding, and daily environmental enrichment. Care staff fed the chimpanzees a diet of commercially produced primate chow, fresh fruits, and vegetables. The chimpanzees also had multiple foraging opportunities every day, and ad libitum access to water.

Subjects were tested in their social groups (18 groups, 2–9 individuals per group, *M* = 5.78, *SD* = 2.27) and ranged in age from 16 to 44 years (*M* = 30.20, *SD* = 5.69). Of the 85 chimpanzees, there were 60 mother-reared and 25 nursery-reared individuals. Wild-born chimpanzees or those with unknown rearing histories (an additional 16 individuals) did participate in the task within their social group, but after examining the differences in the demographics across these groups and considering their unknown early history, we made the decision to exclude these 16 chimpanzees from our analyses. These 16 chimpanzees were mostly female (11 females, 5 males) and older (*M* = 47.56 years old, *SD* = 4.16) than the captive-born population. Wild-born chimpanzees were imported from Africa by other institutions prior to the 1974 CITES importation ban, and those with unknown rearing histories were transferred from other facilities in the 1970s and early 1980s. All work was carried out in accordance with the care and use of animal guidelines as laid out by the National Institutes of Health in the USA and were approved by the Institutional Animal Care and Use Committee at The University of Texas MD Anderson Cancer Center.

All chimpanzees included in the current study had experience with video and picture stimuli through movies and television as part of the enrichment program on site. In addition, they had extensive behavioral testing experience, including participation in cognitive, social learning, training, inequity, and behavioral laterality studies [see citations in 38]. Further, approximately 24% of subjects (n = 19) included in Experiment 1 had been exposed to the touchscreen apparatus used in the current study, having completed training sessions (touching shapes to receive a food reward) for an unrelated study approximately six months prior to the start of this experiment.

### Procedures

**Fig 1:**
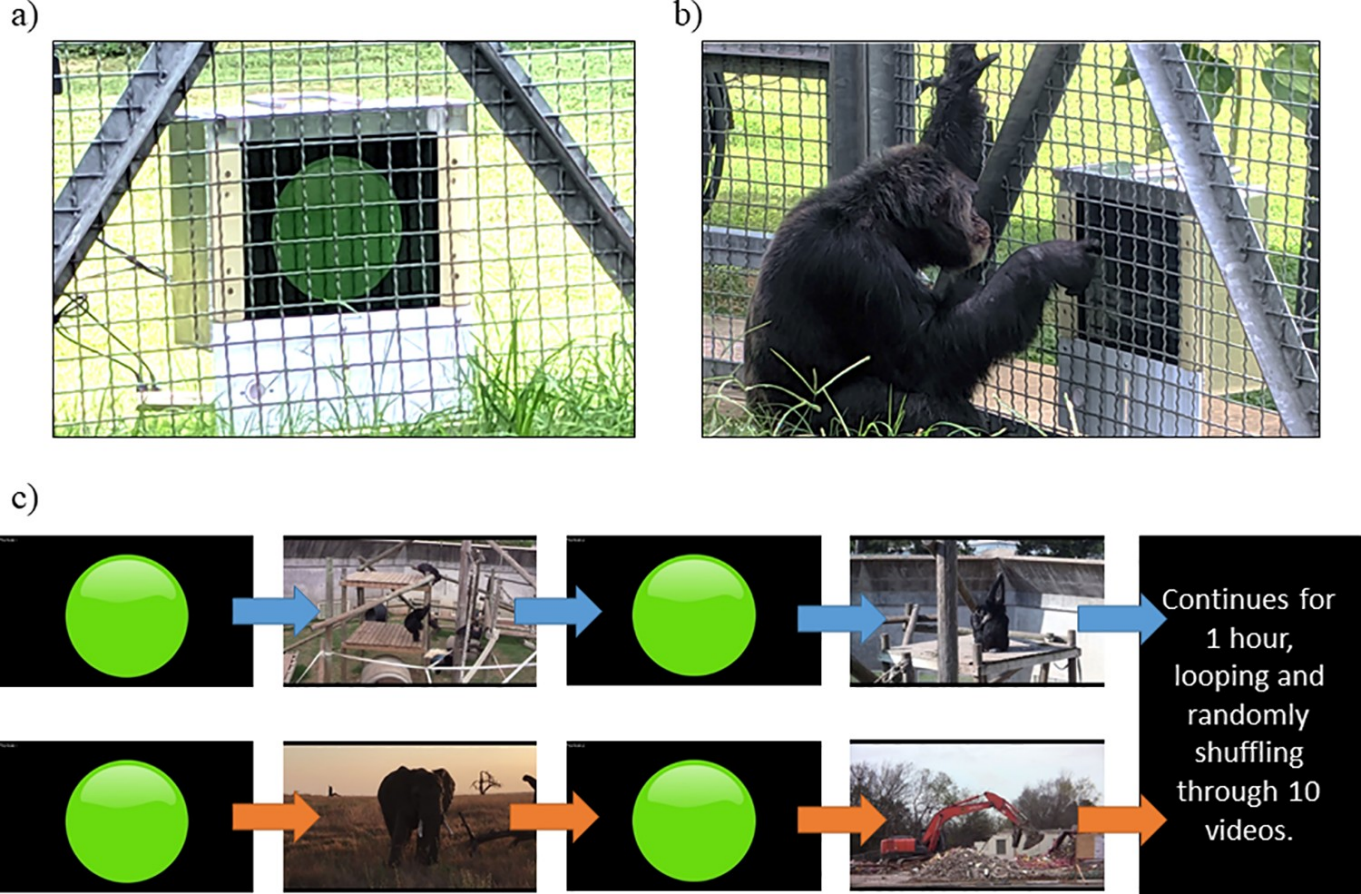
Touchscreen task set-up. a) The apparatus hanging on the wire mesh with the neutral start symbol on the touchscreen. b) A chimpanzee touching the neutral start stimulus to initiate video playback during a trial. c) Examples of how the touch screen program displays social (top) and control (bottom) videos. *Note*: All testing was done in the indoor dens; however, pictures A and B were taken outdoors in order to get a clear view of the screen in use.

Between December 2018 and February 2019, each group of chimpanzees was given access to four, 60-minute test sessions. Each session was separated by at least one day, but no more than three days. During testing, all chimpanzees remained in their social group, had inside/outside access, and free access to food and water. For all sessions, a 19-inch touchscreen (Elo 1991L Open Frame Touchscreen with Accutouch) enclosed in a chimpanzee-proof mesh mount was attached to the enclosure mesh of the inside den (see Fig 1). A researcher controlled the program (run in Java) from a laptop computer connected to the touchscreen.

Each session consisted of ten unique 15-second videos, either social or control, with accompanying audio. Conspecific social videos consisted of familiar (living at the same facility) or unfamiliar (from the wild) chimpanzees (1–8 individuals) engaging in a variety of behaviors, while control videos consisted of moving scenery, vehicles, humans, or other animals engaged in some activity (see S1 Table). Videos in both conditions included audio: although we did not explicitly control for differences in audio between the two conditions, volume was played at the same level across all sessions, and both conditions included a mixture of quiet and loud videos (e.g., chimpanzee screaming in the conspecific social condition and a concert with humans cheering in the control condition).

Each chimpanzee social group was randomly assigned to either the social or control condition first in an ABBA/BAAB design (Fig 1). The subjects initiated a session by touching a neutral start stimulus (a green circle with a 10-inch diameter) in the center of the screen. Once initiated, the chimpanzee was rewarded with a 15-second video clip. This was followed by a 3-second delay in which the screen appeared black, followed by the reappearance of the start stimulus. Chimpanzees could touch the start stimulus to play videos as often as they preferred. After each touch, the program would play one of the 10 videos in random order. Once all 10 videos played, the program would loop and play the videos in random order again (up to 20 times in the 1-hr session). If the subjects did not touch the start stimulus for 2 minutes, the next video would play automatically as a reminder that the task was still available. The two-minute autoplay delay was chosen so that it was short enough to keep their attention on the task (as a reminder that the task was still available), but long enough to allow them ample time to touch the screen if they chose. An experimenter observed each session live and recorded the number of times each chimpanzee in the group initiated video playback by touching the start stimulus. Observers also recorded the number of videos each chimpanzee in the group actively watched as it played (regardless of whether they were the one initiating playback). This was operationally defined as the chimpanzee’s gaze directed toward the screen for a minimum of three consecutive seconds during the video playback, while the chimpanzee was positioned within two meters of the touchscreen. Therefore, a minimum of three seconds of looking at any point during the 15-second trial was needed in order to record the subject as having “looked” at the stimulus, and multiple “looks” were not recorded. The program recorded the timestamp of each touch, the order that the videos played, and noted any videos that played automatically.

As mentioned above, all testing was done within the chimpanzees’ social groups with a single touchscreen apparatus. At the beginning of each session, the apparatus was generally dominated by one or two higher-ranking individuals. However, these individuals did not use the apparatus for the full 60 minutes and tended to leave the testing area once they were done, thus giving lower-ranking individuals a chance to participate. Using multiple touchscreens per session, which would increase access for each group member, was not feasible in the current study [39].

It is also important to note that the three raters in the current experiments were not blind to condition. However, per protocol, raters sat unobtrusively in the testing area and made no attempts to engage with the chimpanzees during the sessions. As such, the touching measure (recorded directly by the touchscreen apparatus) was likely unaffected by potential experimenter bias. Furthermore, we did not explicitly calculate inter-observer reliability when recording which chimpanzees watched or looked at the stimuli during the data collection period. However, the three raters developed and agreed upon operational definitions for dependent variables prior to the study and practiced scoring over two sessions until 100% verbal agreement was achieved.

#### Social cognition and behavior

We also examined archival cognitive and behavioral data from a subset of chimpanzees that participated in Experiment 1. Receptive joint attention was collected in 2011, using procedures originally developed by Dawson et al. [40] and previously utilized with chimpanzees in Hopkins et al. [41]. Briefly, the chimpanzees progressed through two test trials designed to assess the number of social cues needed to elicit an orienting response. To begin, the experimenter would sit in front of the focal chimpanzee and engage them in some type of husbandry behavior. Once the chimpanzee was focused on the experimenter, s/he would end the interaction and look over the subject’s head for 5 seconds before returning to a neutral position. Each trial consisted of three steps with increasing social cues (gaze only, gaze and gesture, and gaze, gesture, and name combined) with associated point values (1, 2, and 3, respectively, and a score of 4 if the chimpanzee never responded). Each trial ended as soon as the chimpanzee oriented in the direction the researcher was looking, and the experimenter recorded the point value [41].

Behavioral observations consisted of 15-minute continuous focal-animal samples collected between July 2016 and May 2018 [42, 43]. Each chimpanzee served as a focal subject in a minimum of 22 observations, although some chimpanzees were observed up to 31 times. We simultaneously recorded proximity to social partners (touching, near, or distant) as well as both social and non-social behaviors [see Neal Webb, Hau [42] for the 51-behavior ethogram used]. For the current study, we examined only proximity, affiliative, submissive, grooming, and anxiety-related behaviors. The composition of the chimpanzee social groups remained stable between the times of behavioral observations (2016–2018) and testing for the current experiments (2019).

### Data analysis

All analyses were conducted in SPSS 24 (IBM, 2018) and significance levels were set at *p* < 0.05 [44]. We calculated the number of videos the chimpanzees initiated and watched in each condition (social and control) and session (1–4). The number of touches and watches in the social and control conditions were positively skewed. However, our within-subjects design and relatively large sample size make these data robust against violations of normality. To examine the relationship between sex and rearing, and the total number of videos the chimpanzees initiated or watched in the social or control condition (that is, one measure representing the sum of all initiated/touched and watched videos across the social and control conditions), we ran independent samples *t*-tests (one with sex as the independent variable and one with rearing as the independent variable). We then ran repeated measures analyses of variance (ANOVAs) with condition (social or control) and session number (1–4) as the repeated independent variables, previous exposure to the touchscreen apparatus (touching a shape to receive a food reward) as the between-subjects factor, and the number of touches to initiate playback or number of videos watched as the dependent variables.

We also examined the relationships between initiating and watching videos on the touchscreen, receptive joint attention (N = 76), and behavior (N = 77). First, we calculated the percentage of trials in which each individual subject initiated playback of social videos [(total number of times chimpanzees touched to play social videos/total number of times they touched to play any videos)*100] and watched social videos [(total number of social videos watched/total number of any videos watched)*100]. For receptive joint attention, we created a composite score that reflected the total number of cues they needed in order to respond (the score of each trial was summed across the two trials). Higher receptive joint attention scores indicated that subjects needed more social cues to elicit a correct orienting response across all trials, and therefore, exhibited poor receptive joint attention skill. Because the data were collected nine years prior to the current experimental data, we were interested in determining if previous performance on the receptive joint attention task was related to future performance (touching and watching videos) on the touchscreen task. For the observational behavioral data, total durations of each behavior (affiliative, submissive, grooming, and anxiety-related behaviors) for each chimpanzee were summed across all observations for that chimpanzee and divided by the total in-view duration to create average durations of behavior. These averages were then converted into percentages [(average behavior duration in seconds/in-view duration in seconds)*100], representing the average percentage of time spent engaged in that behavior. Finally, we used Pearson’s product-moment correlations to examine relationships between scores on the receptive joint attention task and average percentage of time spent engaged in social behaviors (i.e., grooming, affiliative contact, social play, and embrace behaviors, as well as social proximity) and the percentage of videos watched and initiated that had social content.

### Experiment 1 results

#### General touchscreen use

First, across the social and control videos, a total of 95% of subjects (81 of 85) were observed to respond to the start stimulus at least one time, and 100% of subjects were observed to view the videos on at least one occasion. Using independent samples t-tests, there was no significant difference between the use of the touchscreen system and either sex or rearing history of the apes (S2 Table). Recall that each test session was 60 minutes in duration, the videos or pictures were displayed for 15 seconds, and there was a 3-second inter-trial interval. Thus, the maximum number of responses to the touchscreen system for each given group of chimpanzees within a test session was 200. Additionally, recall that if the start stimulus was not touched for 120 seconds, the computer would automatically display a video. Thus, the maximum number of times that video clips could autoplay was 30. The mean numbers of times that the autoplay was activated for social and control video sessions were 12.20 (*SD* = 8.51) and 14.97 (*SD* = 7.20), respectively.

#### Sex and rearing

In the next set of analyses, we tested for the effect of sex and rearing on the number of touches and watching responses across social and control videos. For these analyses, to ensure that we included the chimpanzees that reliably engaged in the touchscreen task, we included only subjects that touched the start stimulus to initiate playback OR watched the videos in a minimum of 10 trials across any condition in the study. After applying these exclusion criteria, the chimpanzee sample consisted of 79 individuals (6 chimpanzees were excluded; 4 mother-reared, 2 nursery-reared; 4 females, 2 males; age M = 32.83, SD = 3.19). We collapsed across touching and watching measures (by summing the number of touches and watches) and ran separate *t*-tests for social and control conditions. These independent samples *t*-tests revealed no sex or rearing differences in the total number of times that chimpanzees interacted with the social or control videos (S2 Table, *p>*0.05); therefore, we collapsed across sex and rearing for all subsequent analyses.

#### Session and order effects

**Fig 2:**
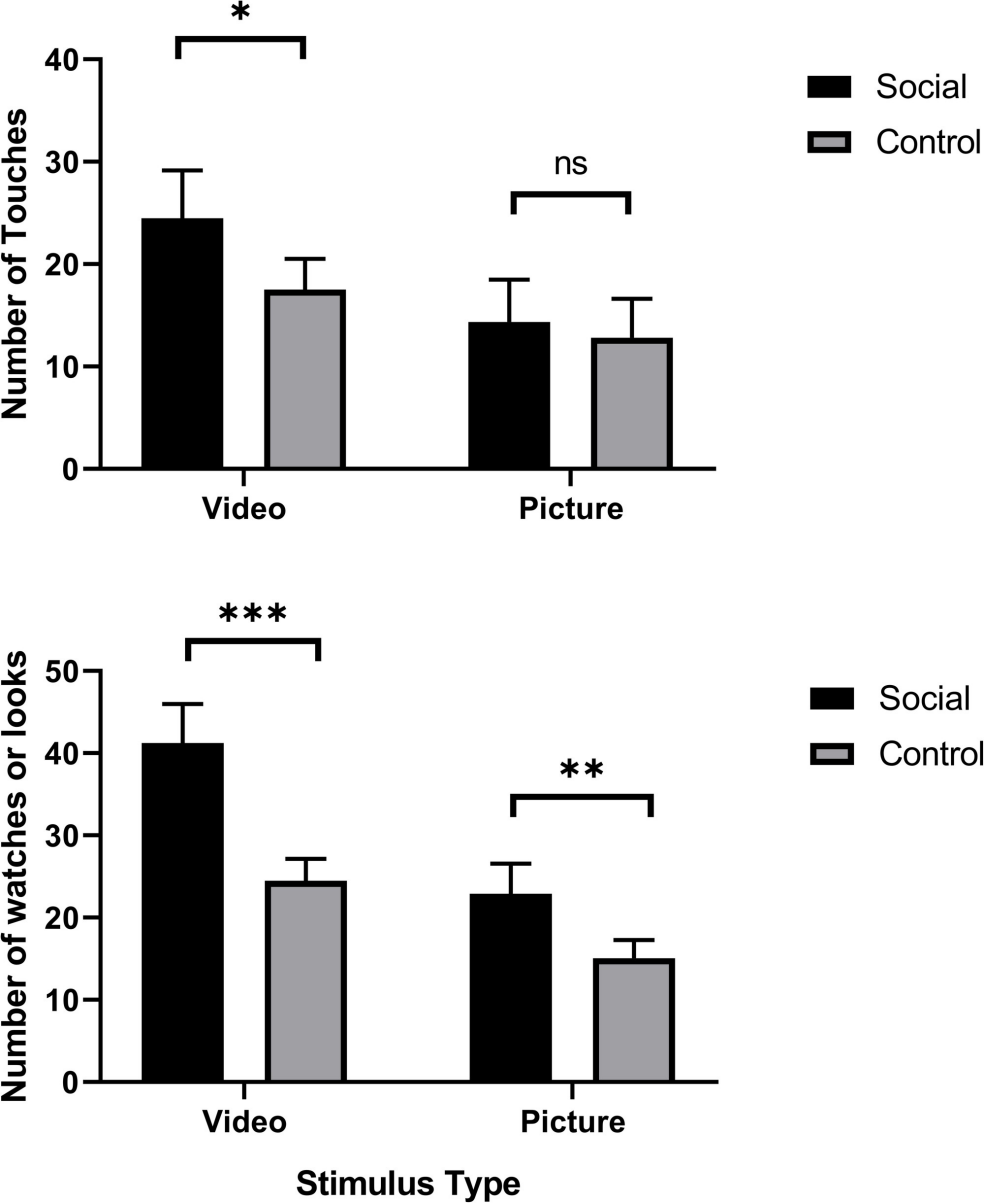
The cumulative number of times the chimpanzees (a) touched to initiate playback of social and control videos and (b) the number of times they watched social and control videos. Bars represent the average across subjects, error bars indicate std. error. *** *p* < 0.001; ** *p* < 0.01; * *p* < 0.05.

**Fig 3:**
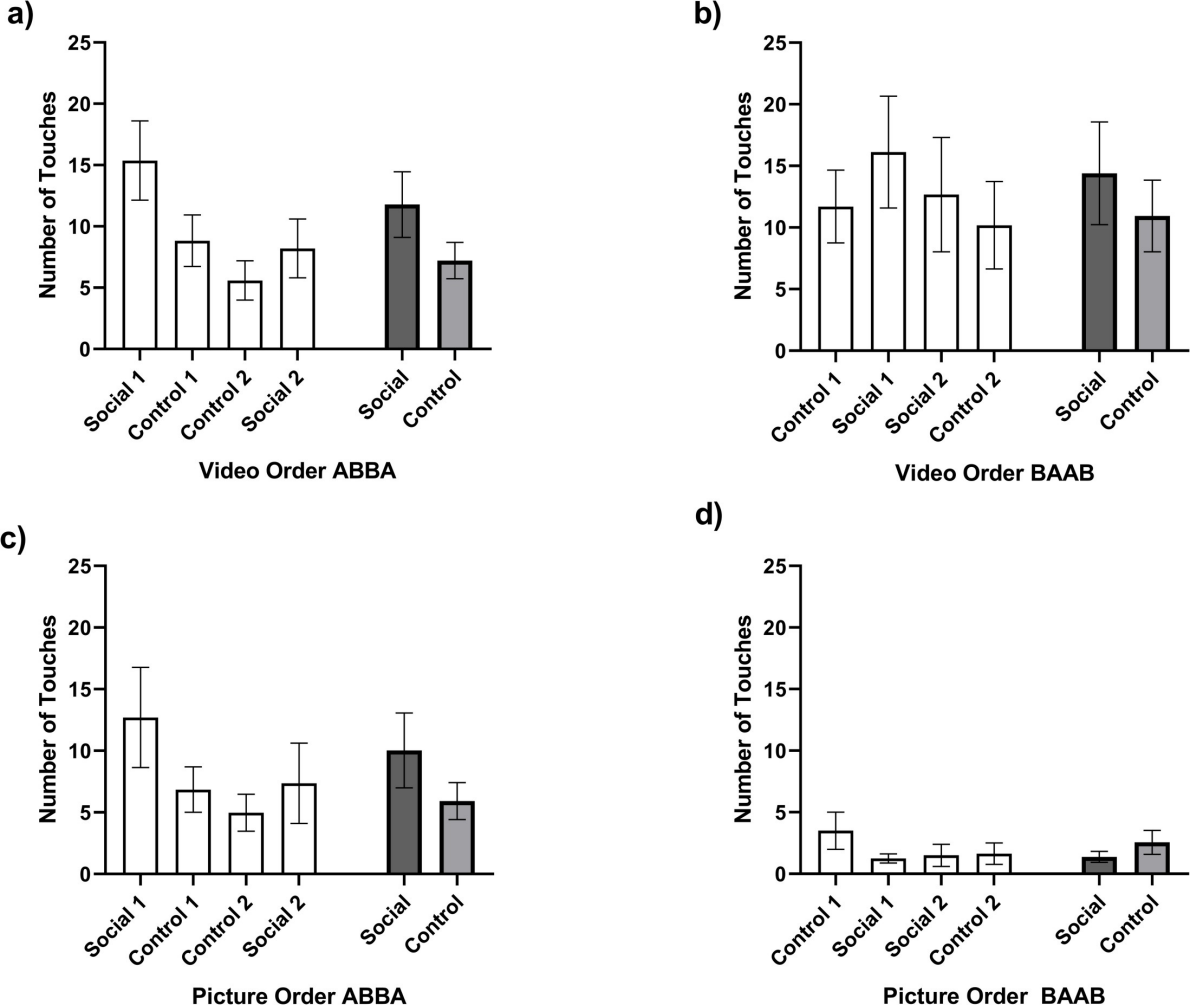
The number of touches (mean ± std. error) during video (a & b) and picture (c & d) experiments as a function of session order (ABBA / BAAB) and condition (social or control). Gray bars represent average number of touches across sessions within each condition.

As mentioned above, 19 subjects had previous exposure to the touchscreen apparatus (touching shapes to receive a food reward). These chimpanzees touched the screen to initiate playback significantly more often than chimpanzees without previous experience in both the social (*M touch with experience* = 20.40, *SE* = 4.67, *M touch without experience* = 10.50, *SE* = 2.63) and control conditions (*M touch with experience* = 13.66, *SE* = 3.00, *M touch without experience* = 7.22, *SE* = 1.69) [*F* (1,77) = 4.07, *p* = 0.047]. However, there was no significant interaction with condition or session [*F* (1,77) = 0.009, *p* = 0.923; *F* (1,77) = 0.78, *p* = 0.381, respectively]. Given that this increased touching for experienced chimpanzees was consistent across conditions and sessions, we continued with the analysis and did not run separate analyses for chimpanzees with and without previous touchscreen experience. The repeated measures analyses of variance (ANOVAs) examining the number of touches to initiate video playback further revealed significant effects of both condition [*F* (1,77) = 6.73, *p* = 0.013] and session [*F* (1,77) = 6.50, *p* = 0.011]. Chimpanzees touched the screen to initiate playback more often during the conspecific social video condition, and the number of touches to initiate playback decreased from session one to session two (within both the social and control conditions; Fig 2). The same was found for watching videos: there was a significant effect of both condition [*F* (1,68) = 14.56, *p* < 0.001], and session [*F* (1,68) = 16.38, *p* < 0.001; Fig 2]. Previous experience with the touchscreen apparatus did not affect the number of videos watched [*F* (1,68) = 0.09, *p* = 0.77], nor did previous experience interact with condition or session [*F* (1,68) = 1.124, *p* = 0.293 and *F* (1,68) = 2.332, *p* = 0.131, respectively]. [Please note that the degrees of freedom for the watching measures is lower than that for the touching measure because we did not have individual data on watching for one group of nine animals during social session 1.] It is worth noting that we found an interesting pattern of response due to session order (see Fig 3). Specifically, when social videos are presented in the first session (i.e., Social 1 within the ABBA order), chimpanzees show a subsequent decrease in responses during the control sessions (Control 1 and Control 2), then increased responding in the final social session (Social 2). When control videos are presented first (Control 1 within the BAAB order), chimpanzees show an increase in responding during the subsequent social session (Social 1), followed by responding behavior decreasing during the remaining sessions (Social 2 and Control 2).

Not all 79 chimpanzees that participated in the touchscreen video task were included in the previous cognitive and behavioral studies, thus reducing the sample size in subsequent analyses (see Table 1). Pearson’s bivariate correlations showed that the percentage of videos watched that had conspecific social content was negatively correlated with receptive joint attention scores (*p*<0.05; Table 1). As higher receptive joint attention scores indicate the chimpanzee required more cues in order to respond, this means that poor receptive joint attention skill was associated with a lower percentage of social videos watched. Receptive joint attention was not correlated with the percentage of videos initiated by touch that had social content (see Table 1).

**Table 1:** 

	Percentage of social videos	
	watched	initiated
Descriptive Statistics	*M* = 58.69%, *SE* = 0.03%	*M* = 55.52%, *SE* = 0.03%
Receptive joint attention	*r*(74) = -0.26, *p =* 0.024, N = 76	*r*(72) = .16, *p* = 0.17, N = 74
Percentage of time spent grooming groupmates	*r*(75) = .10, *p* = 0.37, N = 77	*r*(73) = .23, *p* = 0.05, N = 75
Percentage of time spent engaging in contact affiliative behavior	*r*(75) = .27, *p* = 0.02, N = 77	*r*(73) = -.06, *p* = 0.57, N = 75

Descriptive statistics for percentage of social videos watched and initiated, and correlations between these measures and both social cognition and behavior.

Regarding social behavior, the percentage of videos initiated by touch that had social content was positively correlated with average percentage of time spent grooming groupmates (*p* = 0.05; Table 1). Additionally, we found a positive correlation between the percentage of videos watched that had social content and time spent engaging in contact affiliative behavior (e.g., hand- and mouth-to-body contact) (*p* = 0.02; Table 1). We found no other significant relationships between initiating or watching the social videos and behavior.

## Experiment 2 methods

### Subjects

In order to determine if the results from Experiment 1 would be consistent when using static images, we later (June 2019 –August 2019) used the same paradigm to give the chimpanzees the opportunity to view social or control pictures. Using the same methodology described above, we provided 13 chimpanzee social groups (N = 41, 17 males, 24 females, a subset of the chimpanzees from Experiment 1) with access to social and control pictures. All of these chimpanzees were previously tested using the videos described above. There were 2–8 individuals per group (*M* = 5.15, *SD* = 2.15) and chimpanzees ranged in age from 16 to 39 years (*M* = 30.85, *SD* = 5.63). Of the 41 chimpanzees, there were 29 mother-reared and 12 nursery-reared individuals.

### Procedures

As mentioned above, the chimpanzees were given access to a touchscreen during four, one-hour sessions, each consisting of 10 unique photos displayed for 15 seconds using the same procedures as in Experiment 1. Social pictures consisted of familiar or unfamiliar chimpanzees (1–4 individuals) engaging in a variety of behaviors, while control pictures consisted of scenery, vehicles, or other animals engaged in some activity (S1 Table). Testing using the static images followed all testing with the videos; thus, we did not statistically compare responding between the videos versus static pictures because we did not counterbalance these nor was this a specific hypothesis of interest.

### Data analysis

As was done in Experiment 1, we calculated the number of times the chimpanzees initiated and looked at the pictures in each condition (social and control) and session (1–4). The number of touches and looks in the social and control conditions were positively skewed. However, our within-subjects design and sample size made these data robust against violations of normality. We used the same statistical approach as in Experiment 1. We ran repeated measures analyses of variance (ANOVAs) with condition (social or control) and session number (1–4) as the repeated independent variables, previous exposure to the touchscreen apparatus (touching shapes to receive a food reward) as the between-subjects factor, and the number of touches to initiate picture presentation or number of pictures looked at as the dependent variables.

### Experiment 2 results

#### General touchscreen use

First, across the social and control pictures, 97.5% (40 of 41) and 100% of the subjects were observed to respond to the start stimulus or look at the pictures, respectively. Thus, a very high percentage of the chimpanzees used the touchscreen systems at least once and also looked at the pictures that were presented on the screen. The mean number of times that the autoplay was activated for social and control picture sessions were 17.21 (*SD* = 8.13) and 19.75 (*SD* = 4.47), respectively.

#### Session and order effects

Previous experience with the touchscreen apparatus did not affect touching or looking at the pictures in either the social or control condition [*F* (1,39) = 0.00, *p* = 0.997]. Repeated measures analyses of variance (ANOVAs) examining the number of touches to initiate picture playback revealed no significant effects of condition [*F* (1,39) = 1.82, *p* = 0.19] or session regarding touching to initiate playback [*F*(1,39) = 2.37, *p* = 0.13], but did show significant effects of condition [*F*(1,39) = 7.42, *p* = 0.010] and session [*F* (1,39) = 5.83, *p* = 0.021] regarding the number of times the chimpanzees looked at the pictures (Fig 3). Specifically, chimpanzees looked at more social pictures compared to control pictures, and the number of pictures they looked at decreased from session one to two within both the social and control conditions. Consistent with Experiment 1 using videos, when social pictures were presented in the first session, chimpanzees showed a subsequent decrease in responses during the control sessions, then increased responding in the final social session (see Fig 3). However, when control pictures were presented in the first session (BAAB), overall responding across all subsequent sessions was low (an average of 1–3 touches to initiate display per session).

## Discussion

In Experiment 1, chimpanzees initiated the playback of more conspecific social videos than control videos and watched the conspecific videos more than control videos. Furthermore, receptive joint attention performance (i.e., social cognition) was correlated with the percentage of videos watched that had conspecific social content. Lastly, chimpanzees that engaged in more contact affiliative behavior (i.e., social behavior) watched a higher number of social videos. However, the percentage of videos initiated with social content was not associated with receptive joint attention but was associated with the amount of grooming given to social partners. Overall, our results indicate that chimpanzees are socially motivated, with conspecific social content being more rewarding and garnering more attention than control content. Unlike previous research with rhesus macaques which showed that only two monkeys ever learned to press a lever to play conspecific videos [23], chimpanzees increased touchscreen presses when rewarded with conspecific videos (without any explicit training on this task). Furthermore, this social motivation is positively associated with both social behavior and social cognition. Finally, when testing with static images in Experiment 2, we found that chimpanzees looked at the social pictures of other chimpanzees more than control pictures, but they did not touch the screen to initiate presentation of social pictures significantly more often than control pictures. It is worth noting that chimpanzees’ responses to the static stimuli in Experiment 2 were overall lower than responses in Experiment 1, likely suggesting that picture stimuli were less rewarding than the video stimuli.

The social motivation theory posits that there are three components of social motivation: 1) social orienting, or attention toward social stimuli; 2) social reward, or seeking out social stimuli due to their inherently rewarding nature; and 3) social maintaining, whereby individuals expend energy to engage with others in order to establish, maintain, and enhance relationships [1]. In the current study, the attentional bias toward conspecific social content (watching) was associated with social behavior and social cognition, as predicted by the social motivation theory [1]. Those with higher attention toward social videos also had higher levels of contact affiliative behavior, behaviors that serve as a mechanism by which chimpanzees establish, maintain, and enhance relationships with their groupmates. Additionally, chimpanzees that were better at receiving and responding to joint attention cues years earlier showed a higher level of social attention (higher percentage of social videos watched), thus demonstrating that the ability to receive and respond to social cues was related to social attention almost a decade later. This is consistent with findings in human clinical populations. Children with Autism Spectrum Disorder (ASD), who exhibit lower social motivation, show deficits in receptive joint attention, likely due to reductions in social attention: a child must attend to social stimuli in order to interpret those cues [1, 45]. Lastly, we believe touching the screen to initiate a video with conspecific social content may correspond to the social reward component of social motivation; that is, the chimpanzees exerted more effort (touched the screen) in order to obtain the conspecific social reward (i.e. videos of conspecifics) than controls. This social reward was correlated with time spent grooming groupmates, which, similar to contact affiliative behavior, is an important social maintenance behavior for chimpanzees. Therefore, the relationships found in the current study, among social attention, cognition, and behavior are, overall, quite consistent with the social motivation theory.

Previous research suggests that even static images may be intrinsically rewarding [4]; however the current study showed that although the chimpanzees looked at the screen more when social pictures were displayed, they did not touch the screen to initiate the display of social pictures any more than control pictures. Studies of human populations suggest that static images are less salient than dynamic video stimuli [1, 46, 47]. The increased initiation of conspecific social videos in our first study was not replicated in the second study with the social pictures. This may indicate that the static social images were less rewarding than the dynamic videos. Alternatively, it is possible that the lower rate of responding to the pictures was a result of the decreased novelty of the touchscreen apparatus itself. We did not counterbalance the picture and video experiments, and as such, all subjects were exposed to the pictures at least six months after the conclusion of the video experiment. Therefore, given that subjects had been previously exposed to these procedures, it is possible that they were less interested in the task as a whole. However, this seems unlikely, as our data show that those who had previous experience with the touchscreen actually touched the screen more in Experiment 1. Additionally, given that six months elapsed between the two experiments, we believe that habituation to the task was unlikely to have occurred. As such, we believe that the most parsimonious explanation is that the static images themselves were simply less rewarding. Because there was no direct comparison between responses to picture and video rewards, future research should modify the current touchscreen task to directly compare the differences in chimpanzee attention and the strength of dynamic and static social rewards.

In addition, we did not assess possible relationships between chimpanzees’ orientation toward humans and the measures used in the current study. The joint attention task involved a human experimenter giving cues to the chimpanzees. It is possible that some chimpanzees perform better on these tasks than others because they are more human-oriented in general. Additionally, some of the videos in the control category included humans (e.g., a child dancing). This represents a confound in the current study; however, our results showed that chimpanzees expended more effort to play conspecific social videos (and thus, these videos were likely better reinforcers/rewards) than the control videos, even with the inclusion of humans. Because some chimpanzees have human-oriented biases (regardless of rearing status), it is possible that the difference between the number of watches and touches during the social and control video conditions would have been even more pronounced if the control videos had included only inanimate objects or non-human animals. Future research should examine the ways that human orientation impacts performance on social communication measures, social reward value, and social behavior of captive chimpanzees.

We found in Experiment 1 that chimpanzees that had previous experience with the touchscreen apparatus (through training sessions to touch shapes on the screen) touched the screen to play social and control videos more than those without previous experience. This is likely due to familiarity with how the apparatus functioned; that is, the chimpanzees understood that touching the screen elicits a response from the apparatus. This is supported by the finding that the frequency of watching videos in Experiment 1, as well as initiating and looking at pictures in Experiment 2, did not differ with previous touchscreen experience. At the start of Experiment 2, all chimpanzees technically had previous touchscreen experience due to their participation in Experiment 1, and indeed we found no difference in frequency of responding in Experiment 2. In fact, the same pattern of increased touching to initiate playback of social rewards emerged in both experienced and non-experienced subjects, further supporting the idea that social content is innately rewarding (although, again, it is noteworthy that touching was still very low overall in Experiment 2).

Providing visual stimulation via television is a common form of enrichment for captive nonhuman primates, including macaques, baboons, and apes [11, 48, 49]. To our knowledge, just one study has examined the behavioral effects of different types of video enrichment in chimpanzees (but see [50] for video enrichment in four gorillas). Bloomsmith & Lambeth [19] presented chimpanzees with videos of conspecifics, animals and humans, and television programs, and found sustained attention to the monitor during playback of a video of humans and chimpanzees interacting compared to a blank screen [19]. Additionally, as mentioned above, there is very limited evidence that nonhuman primates would choose to play videos to watch if given a choice [11, 12]. The results from the current study explicitly demonstrate the difference in reward value between social and control video and picture stimuli. Our results suggest that videos of known and unknown conspecifics are inherently rewarding. Additionally, videos of humans, non-primate animals, and inanimate objects are somewhat rewarding, but are certainly less rewarding than those that include conspecifics. Overall, we suggest that chimpanzees will choose to interact with a touchscreen that presents video stimuli, and that they find conspecific social content more rewarding than other content, thus confirming the enrichment value of social video stimulation.

In addition, we found an interesting pattern of responding as a function of session order (Fig 3). Chimpanzees in the ABBA order (i.e., those that started the experiment with social videos) showed a high rate of touches and watches in their first session, a social session. Their responses decreased during their second and third sessions (control sessions), and then showed a slight increase when social rewards were presented again in the fourth session. This was true for both the video and picture presentations. This decreased responding to obtain control content and increased responding at the return of social content adds to the argument that social content is rewarding—chimpanzees increased touchscreen responses when rewarded with conspecific social content following lower rates of responding when rewarded with control content. Similarly, chimpanzees in the BAAB order showed increased touching responses in session 2, when social rewards were presented after the first control session (although this was not the case with the watching measure). Again, this seems to add to the argument that social videos are rewarding. However, it should also be noted that both touching and watching responses decreased from the first to the second social reward session, perhaps suggesting some habituation after the first one-hour session. Regarding picture rewards, in order BAAB chimpanzees showed a higher number of responses in the first session (a control reward session), followed by an extremely low rate of responding in all remaining sessions (less than one touch per session). The novelty of the control stimuli upon first presentation in the picture stimuli resulted in higher responding than subsequent sessions. However, the overall low rate of responding across the entire BAAB order (only 1–3 touches per session) may suggest that static images are likely not reinforcing at all compared to dynamic, video rewards.

Several additional lines of research are possible to further examine the relationships among social motivation, cognition, and behavior. For example, it is unknown whether social attention can be increased through training. This would have implications for ASD interventions with children, perhaps increasing social attention at an early age, thereby increasing opportunities for social interactions and learning. Additionally, future research should examine the underlying neurological mechanisms related to the variation in social motivation reported herein. Several brain regions, such as the orbitofrontal-striatum-amygdala network, nucleus accumbens, ventral striatum, and the ventral tegmental area, have been associated with increased social orientation, as well as pursuing and processing social reward in humans [51–54]. In addition, the posterior superior temporal gyrus has been linked to poor socio-communicative abilities in human clinical populations [e.g. those diagnosed with schizophrenia or autism spectrum disorders; 55–57], which has also been demonstrated in chimpanzees. Chimpanzees with poor receptive joint attention abilities had less gray matter in the right posterior superior temporal gyrus compared to those who performed well [41]. Additional research is needed to determine if similar brain regions are associated with performance on the touchscreen task used in the current study (using archival brain imaging data from chimpanzees; or collecting new data, both touchscreen and brain imaging with other nonhuman primate species). Finally, monkeys that received intranasal oxytocin showed an increase in both time spent viewing and gaze following saccades when presented with conspecific social videos, providing evidence that oxytocin increases social motivation in primates [13]. Additional research is needed to determine if chimpanzees with higher social motivation have either increased oxytocin levels, or different oxytocin receptor genotypes or gene expression.

In summary, we show that chimpanzees find social stimuli inherently rewarding and seek out opportunities to engage with such stimuli. Furthermore, this inherent social motivation is associated with both social cognition and behavior, consistent with the social motivation theory. Future research regarding the individual variability in these relationships, as well as underlying mechanisms, may prove useful in understanding typical variation in both human and nonhuman primate social behavior.

## Supporting information

S1 Table Description of social and nonsocial video and picture rewards used in experiments. (DOCX)

S2 Table Non-significant results for Experiment 1. (XLSX)

S1 Data Data file containing the individual data from Experiment 1 and 2, and the group autoplay data. (XLSX)

## References

[1] Chevallier C, Kohls G, Troiani V, Brodkin ES, Schultz RT. The social motivation theory of autism. Trends Cogn Sci. 2012;16(4):231–9. Epub 2012/03/20. doi: 10.1016/j.tics.2012.02.007 ; PubMed Central PMCID: PMC3329932. 22425667 PMC3329932

[2] Salva OR, Mayer U, Vallortigara G. Roots of a social brain: Developmental models of emerging animacy-detection mechanisms. Neuroscience & Biobehavioral Reviews. 2015;50:150–68. 25544151 10.1016/j.neubiorev.2014.12.015

[3] Salva OR, Farroni T, Regolin L, Vallortigara G, Johnson MH. The evolution of social orienting: evidence from chicks (Gallus gallus) and human newborns. PloS one. 2011;6(4):e18802. doi: 10.1371/journal.pone.0018802 21533093 PMC3080385

[4] Anderson JR. Social stimuli and social rewards in primate learning and cognition. Behavioural Processes. 1998;42(2):159–75. doi: 10.1016/s0376-6357(97)00074-0 24897460

[5] Andrews MW, Rosenblum LA. Live-social-video reward maintains joystick task performance in bonnet macaques. Perceptual and Motor Skills. 1993;77(3):755–63. doi: 10.2466/pms.1993.77.3.755 8284149

[6] Campbell MW, Carter JD, Proctor D, Eisenberg ML, de Waal FB. Computer animations stimulate contagious yawning in chimpanzees. Proceedings of the Royal Society B: Biological Sciences. 2009;276(1676):4255–9. doi: 10.1098/rspb.2009.1087 19740888 PMC2821339

[7] Bard KA, Platzman KA, Lester BM, Suomi SJ. Orientation to social and nonsocial stimuli in neonatal chimpanzees and humans. Infant Behavior and Development. 1992;15(1):43–56. doi: 10.1016/0163-6383(92)90005-q.

[8] Boccia ML, Reite M, Laudenslager M. On the physiology of grooming in a pigtail macaque. Physiology & Behavior. 1989;45(3):667–70. 2756061 10.1016/0031-9384(89)90089-9

[9] Dunbar RI. The social role of touch in humans and primates: Behavioural function and neurobiological mechanisms. Neuroscience & Biobehavioral Reviews. 2010;34(2):260–8. doi: 10.1016/j.neubiorev.2008.07.001 18662717

[10] Fagot J. Picture perception in animals. Sussex: Psychology Press; 2000.

[11] Ogura T. Use of video system and its effects on abnormal behaviour in captive Japanese macaques (*Macaca fuscata*). Applied animal behaviour science. 2012;141(3–4):173–83.

[12] Ogura T, Matsuzawa T. Video preference assessment and behavioral management of single-caged Japanese macaques (*Macaca fuscata*) by movie presentation. Journal of applied animal welfare science. 2012;15(2):101–12. doi: 10.1080/10888705.2012.624887 22458872

[13] Putnam PT, Roman JM, Zimmerman PE, Gothard KM. Oxytocin enhances gaze-following responses to videos of natural social behavior in adult male rhesus monkeys. Psychoneuroendocrinology. 2016;72:47–53. Epub 2016/06/28. doi: 10.1016/j.psyneuen.2016.05.016 ; PubMed Central PMCID: PMC5226068. 27343726 PMC5226068

[14] Rossion B, Taubert J. What can we learn about human individual face recognition from experimental studies in monkeys? Vision Res. 2019;157:142–58. Epub 2019/06/25. doi: 10.1016/j.visres.2018.03.012 . 31230664

[15] Machado CJ, Bliss-Moreau E, Platt ML, Amaral DG. Social and nonsocial content differentially modulates visual attention and autonomic arousal in Rhesus macaques. PLoS One. 2011;6(10):e26598. Epub 2011/11/03. doi: 10.1371/journal.pone.0026598 ; PubMed Central PMCID: PMC3202553. 22046313 PMC3202553

[16] Hopper LM, Lambeth SP, Schapiro SJ. An evaluation of the efficacy of video displays for use with chimpanzees (*Pan troglodytes*). American Journal of Primatology. 2012;74(5):442–9. doi: 10.1002/ajp.22001 22318867 PMC3823527

[17] Howard LH, Wagner KE, Woodward AL, Ross SR, Hopper LM. Social models enhance apes’ memory for novel events. Scientific reports. 2017;7(1):1–7. doi: 10.1038/s41598-016-0028-x 28106098 PMC5247682

[18] Brent L, Stone AM. Long-term use of televisions, balls, and mirrors as enrichment for paired and singly caged chimpanzees. American Journal of Primatology. 1996;39(2):139–45. Epub 1996/01/01. doi: 10.1002/(SICI)1098-2345(1996)39:2&lt;139::AID-AJP5&gt;3.0.CO;2-# . 31918497

[19] Bloomsmith M, Lambeth S. Videotapes as enrichment for captive chimpanzees. Zoo Biology. 2000;19:541–51. doi: 10.1002/1098-2361(2000)19:6&lt;541::AID-ZOO6&gt;3.0.CO;2-3 11180415

[20] Wells DL. Sensory stimulation as environmental enrichment for captive animals: A review. Applied Animal Behaviour Science. 2009;118(1–2):1–11. doi: 10.1016/j.applanim.2009.01.002

[21] Andrews MW, Rosenblum LA. Response patterns of bonnet macaques following up to 75 weeks of continuous access to social‐video and food rewards. American Journal of Primatology. 2002;57(4):213–8. doi: 10.1002/ajp.10044 12210673

[22] Washburn DA, Hopkins WD. Videotape-versus pellet-reward preferences in joystick tasks by macaques. Perceptual and motor skills. 1994;78(1):48–50. doi: 10.2466/pms.1994.78.1.48 8177686

[23] Harris LD, Briand EJ, Orth R, Galbicka A. Assessing the value of television as environmental enrichment for individually housed rhesus monkeys: A behavioral economic approach. Journal of the American Association for Laboratory Animal Science. 1999;38(2):48–53. 12086433

[24] Gray H, Pearce B, Thiele A, Rowe C. The use of preferred social stimuli as rewards for rhesus macaques in behavioural neuroscience. PLoS One. 2017;12(5):e0178048. Epub 2017/05/26. doi: 10.1371/journal.pone.0178048 ; PubMed Central PMCID: PMC5444662. 28542356 PMC5444662

[25] Leavens DA. Joint attention: Twelve myths. In: Seemann A, editor. Joint attention: New developments in psychology, philosophy of mind and social neuroscience. Cambridge, MA: MIT Press; 2012. p. 43–72.

[26] Leavens DA, Bard KA. Environmental influences on joint attention in great apes: Implications for human cognition. Journal of Cognitive Education and Psychology. 2011;10:9–31.

[27] Leavens DA, Bard KA, Hopkins WD. The mismeasure of ape social cognition. Anim Cogn. 2017. doi: 10.1007/s10071-017-1119-1 28779278 PMC6647540

[28] Clark H, Elsherif MM, Leavens DA. Ontogeny vs. phylogeny in primate/canid comparisons: A meta-analysis of the object choice task. Neurosci Biobehav Rev. 2019;105:178–89. Epub 2019/06/07. doi: 10.1016/j.neubiorev.2019.06.001 . 31170434

[29] Liszkowski U, Carpenter M, Henning A, Striano T, Tomasello M. Twelve-month-olds point to share attention and interest. Dev Sci. 2004;7(3):297–307. Epub 2004/12/15. doi: 10.1111/j.1467-7687.2004.00349.x . 15595371

[30] Tomasello M. Origins of human communication. Cambridge, MA: MIT Press; 2008.

[31] Leavens DA, Hopkins WD, Bard KA. Understanding the Point of Chimpanzee Pointing: Epigenesis and Ecological Validity. Curr Dir Psychol Sci. 2005;14(4):185–9. Epub 2007/12/27. doi: 10.1111/j.0963-7214.2005.00361.x ; PubMed Central PMCID: PMC2151757. 18159225 PMC2151757

[32] Bottini S. Social reward processing in individuals with autism spectrum disorder: A systematic review of the social motivation hypothesis. Research in Autism Spectrum Disorder. 2018;45:9–26.

[33] Tomasello M. The cultural origins of human cognition. Cambridge, MA: Harvard University Press; 2000.

[34] Hopkins WD, Misiura M, Reamer LA, Schaeffer JA, Mareno MC, Schapiro SJ. Poor receptive joint attention skills are associated with atypical gray matter asymmetry in the posterior superior temporal gyrus of chimpanzees (*Pan troglodytes*). Frontiers in Psychology. 2014;5. doi: 10.3389/fpsyg.2014.00007 24523703 PMC3905213

[35] Skinner B. The behavior of organisms: An experimental analysis. New York: D. Appleton-Century Company nc.; 1938.

[36] Sackett GP, Ruppenthal GC, Elias K. Nursery rearing of nonhuman primates in the 21st century. Tuttle R, H, editor. New York, NY: Springer Science+Business Media; 2006.

[37] Russell CL, Bard KA, Adamson LB. Social referencing by young chimpanzees (*Pan troglodytes*). Journal of Comparative Psychology. 1997;111:185–93. doi: 10.1037/0735-7036.111.2.185 9170283

[38] Neal Webb SJ, Hau J, Schapiro SJ. Relationships between captive chimpanzee (*Pan troglodytes*) welfare and voluntary participation in behavioural studies. Applied animal behaviour science. 2019;214:102–9. doi: 10.1016/j.applanim.2019.03.002 31244501 PMC6594403

[39] Cronin KA, Jacobson SL, Bonnie KE, Hopper LM. Studying primate cognition in a social setting to improve validity and welfare: A literature review highlighting successful approaches. PeerJ. 2017;5:e3649. doi: 10.7717/peerj.3649 28791199 PMC5545107

[40] Dawson G, Munson J, Estes A, Osterling J, McPartland J, Toth K, et al. Neurocognitive function and joint attention ability in young children with autism spectrum disorder versus developmental delay. Child Development 2002;73(2):345–58. doi: 10.1111/1467-8624.00411 11949896

[41] Hopkins W, Misiura M, Reamer L, Schaeffer J, Mareno MC, Schapiro S. Poor receptive joint attention skills are associated with atypical gray matter asymmetry in the posterior superior temporal gyrus of chimpanzees (*Pan troglodytes*). Frontiers in Psychology. 2014;5(7). doi: 10.3389/fpsyg.2014.00007 24523703 PMC3905213

[42] Neal Webb SJ, Hau J, Schapiro SJ. Captive chimpanzee (*Pan troglodytes*) behavior as a function of space per animal and enclosure type. American journal of primatology. 2018;80(3):e22749. doi: 10.1002/ajp.22749 29575053 PMC6472486

[43] Neal Webb SJ, Hau J, Schapiro SJ. Does group size matter? Captive chimpanzee (*Pan troglodytes*) behavior as a function of group size and composition. American journal of primatology. 2019;81(1):e22947. doi: 10.1002/ajp.22947 30620093 PMC6472487

[44] Perenger TV. What’s wrong with Bonferroni adjustments. British Medical Journal. 1998;316(7139):1236–8. doi: 10.1136/bmj.316.7139.1236 9553006 PMC1112991

[45] Mundy P. A review of joint attention and social-cognitive brain systems in typical development and autism spectrum disorder. European Journal of Neuroscience. 2017;47(6):497–514. doi: 10.1111/ejn.13720 28922520

[46] Chevallier C, Parish-Morris J, McVey A, Rump KM, Sasson NJ, Herrington JD, et al. Measuring social attention and motivation in autism spectrum disorder using eye-tracking: Stimulus type matters. Autism Research. 2015;8(5):620–8. doi: 10.1002/aur.1479 26069030 PMC4624010

[47] Kohls G, Chevallier C, Troiani V, Schultz RT. Social ’wanting’ dysfunction in autism: Neurobiological underpinnings and treatment implications. J Neurodev Disord. 2012;4(1):10. Epub 2012/09/11. doi: 10.1186/1866-1955-4-10 ; PubMed Central PMCID: PMC3436671. 22958468 PMC3436671

[48] Platt DM, Novak MA. Videostimulation as enrichment for captive rhesus monkeys (*Macaca mulatta*). Applied Animal Behaviour Science. 1997;52:139–55.

[49] Schapiro SJ. Handbook of primate behavioral management: CRC Press; 2017.

[50] Maloney MA, Leighty KA, Kuhar CW, Bettinger TL. Behavioral responses of silverback gorillas (*Gorilla gorilla gorilla*) to videos. Journal of applied animal welfare science. 2011;14(2):96–108. doi: 10.1080/10888705.2011.551621 21442506

[51] Hung LW, Neuner S, Polepalli JS, Beier KT, Wright M, Walsh JJ, et al. Gating of social reward by oxytocin in the ventral tegmental area. Science. 2017;357(6358):1406. doi: 10.1126/science.aan4994 28963257 PMC6214365

[52] Kohls G, Perino MT, Taylor JM, Madva EN, Cayless SJ, Troiani V, et al. The nucleus accumbens is involved in both the pursuit of social reward and the avoidance of social punishment. Neuropsychologia. 2013;51(11):2062–9. doi: 10.1016/j.neuropsychologia.2013.07.020 23911778 PMC3799969

[53] Kohls G, Schulte-Rüther M, Nehrkorn B, Müller K, Fink GR, Kamp-Becker I, et al. Reward system dysfunction in autism spectrum disorders. Social Cognitive and Affective Neuroscience. 2012;8(5):565–72. doi: 10.1093/scan/nss033 22419119 PMC3682440

[54] Richey JA, Rittenberg A, Hughes L, Damiano CR, Sabatino A, Miller S, et al. Common and distinct neural features of social and non-social reward processing in autism and social anxiety disorder. Social Cognitive and Affective Neuroscience. 2013;9(3):367–77. doi: 10.1093/scan/nss146 23223206 PMC3980795

[55] Boddaert N, Chabane N, Gervais H, Good CD, Bourgeois M, Plumet M-H, et al. Superior temporal sulcus anatomical abnormalities in childhood autism: A voxel-based morphometry MRI study. NeuroImage. 2004;23:364–9. doi: 10.1016/j.neuroimage.2004.06.016 15325384

[56] Sommer I, Ramsey N, Kahn R. Handedness, language lateralisation and anatomical asymmetry in schizophrenia: Meta-analysis. British Journal of Psychiatry. 2001;178:344–51. 10.1192/bjp.178.4.344 11282814

[57] Zilbovicius M, Meresse I, Chabane N, Brunelle F, Samson Y, Boddaert N. Autism, the superior temporal sulcus and social perception. Trends in Neurosciences. 2006;29(7):359–66. doi: 10.1016/j.tins.2006.06.004 16806505

