# Supplementary material for: Are conspecific social videos rewarding to chimpanzees (Pan troglodytes)? A test of the social motivation theory
Source: PLoS One. 2021 Nov 24;16(11):e0259941. doi: 10.1371/journal.pone.0259941 (PMC8612576; doi:10.1371/journal.pone.0259941)
Supplement: S1 Table — (DOCX) [file pone.0259941.s001.docx]

| **S1 Table.** **Description of social and nonsocial video and picture rewards used in experiments.** | | | |
| --- | --- | --- | --- |
| Condition | Description | Condition | Description |
| Social Videos | Unknown wild chimpanzee group vocalizing and travelling | Social Pictures | One KCCMR adult chimpanzee looking toward camera |
|  | Unknown wild chimpanzee group vocalizing and travelling with narration |  | Two KCCMR adult chimpanzees looking toward camera |
|  | Two KCCMR adult chimpanzees eating pumpkin |  | One wild, unknown, adult chimpanzee walking bipedally |
|  | One unknown wild chimpanzee display and scream |  | One KCCMR adult chimpanzee looking toward camera |
|  | KCCMR chimpanzee group during agonistic encounter |  | One KCCMR adult chimpanzees resting |
|  | KCCMR chimpanzee group receiving enrichment |  | Three KCCMR adult chimpanzees sitting alert |
|  | One KCCMR chimpanzee following another chimpanzee |  | Five KCCMR adult chimpanzees sitting, walking, hanging |
|  | Two KCCMR chimpanzees hand-clasp grooming |  | One KCCMR adult chimpanzee looking toward camera |
|  | One unknown wild chimpanzee walking in forest |  | Two KCCMR adult chimpanzees grooming |
|  | One unknown wild chimpanzee sitting in forest |  | One KCCMR adult chimpanzee looking toward camera |
|  | Unknown, wild chimpanzee group during agonistic encounter |  | One KCCMR adult chimpanzee looking toward camera |
|  | Two KCCMR chimpanzees playing |  | One KCCMR adult chimpanzee looking toward camera |
|  | One KCCMR chimpanzee laying in nest |  | Two KCCMR juvenile chimpanzees playing |
|  | One KCCMR chimpanzee nesting |  | Two KCCMR adult chimpanzees sitting |
|  | Two KCCMR chimpanzees playing chase |  | One KCCMR adult chimpanzee looking toward camera |
|  | KCCMR chimpanzee group receiving presents |  | Two KCCMR adult chimpanzees sitting |
|  | KCCMR chimpanzees engaging in group grooming |  | Two KCCMR adult chimpanzees with tumescent swellings |
|  | Two KCCMR chimpanzees grooming each other |  | One KCCMR adult chimpanzee looking toward camera with play face |
|  | Two KCCMR chimpanzees, one starting display (swaying) |  | One KCCMR adult chimpanzee looking toward camera with play face |
|  | One wild, unknown chimpanzee carrying multiple food items through forest |  | One KCCMR adult chimpanzee looking toward camera |
| Non-social Videos | One human baby vocalizing | Non-social Pictures | Mountain scenery with fog |
|  | Kite surfers on Australian beach |  | Albino peacock plume |
|  | Buffalo herd foraging |  | Downtown street front |
|  | Female longhorn walking |  | Australian beach |
|  | Female longhorn digging |  | Trees and bridge |
|  | Building teardown with hydraulic excavator |  | Two water buffalo walking |
|  | Human toddler dancing |  | Flowers and bush |
|  | One elephant walking in grassland |  | Chameleon walking on sidewalk |
|  | Elephant herd walking across road |  | Three elephants walking on grass |
|  | Flags waving in wind |  | Flowers |
|  | Two human toddlers talking and laughing with baby |  | Gazelle herd foraging |
|  | Thousands of humans marching in street |  | One giraffe face |
|  | Human dressed as penguin dancing with music |  | Two domestic cats sitting |
|  | Cars racing |  | Moth on foliage |
|  | River flowing |  | Porch |
|  | Two elephants standing |  | Ship in water |
|  | First-person view of driving through trees |  | Storm clouds |
|  | Rap concert |  | Thistle in grass |
|  | Tesla coils playing Bohemian Rhapsody |  | Vehicle on safari |
|  | Three human dancing performers with band |  | Two Zebra foraging |
| *Note*: In the social condition, videos were chosen with the goal of including various unfamiliar (from the wild) and familiar (from KCCMR facility) chimpanzees engaging in a wide range of behaviors. In the control condition, videos were chosen with the goal of including a mix of videos with scenery, animate (humans and non-primate animals) and inanimate moving objects. Comparable videos were chosen within the social and control conditions; that is, similar videos between social session 1 and social session 2, and between control session 1 and control session 2 | | | |
